# A Comparative Study on the Effect of Acute Pharyngeal Stimulation with TRP Agonists on the Biomechanics and Neurophysiology of Swallow Response in Patients with Oropharyngeal Dysphagia

**DOI:** 10.3390/ijms231810773

**Published:** 2022-09-15

**Authors:** Noemí Tomsen, Omar Ortega, Daniel Alvarez-Berdugo, Laia Rofes, Pere Clavé

**Affiliations:** 1 Gastrointestinal Physiology Laboratory, Hospital de Mataró, Consorci Sanitari del Maresme, 08304 Mataró, Spain; 2 Centro de Investigación Biomédica en Red de Enfermedades Hepáticas y Digestivas, Instituto de Salud Carlos III, 28029 Barcelona, Spain

**Keywords:** deglutition disorders, gastroenterology, therapeutics, transient receptor potential channels, sensory stimulation, swallowing function

## Abstract

Fluid thickening is the main compensatory strategy for patients with oropharyngeal dysphagia (OD) associated with aging or neurological diseases, and there is still no pharmacological treatment. We aimed to compare the effects of increasing bolus viscosity with that of acute stimulation with TRPV1, TRPA1 or TRPM8 agonists on the biomechanics and neurophysiology of swallow response in patients with OD. We retrospectively analyzed seven studies from our laboratory on 329 patients with OD. The effect of increasing shear viscosity up to 3682 mPa·s was compared by videofluoroscopy and pharyngeal sensory evoked potentials (pSEP) with that of adding to the bolus: capsaicin (TRPV1, 150 μM/10 μM), piperine (TRPA1/V1, 1 mM/150 μM), menthol (TRPM8, 1 mM/10 mM), cinnamaldehyde-zinc (TRPA1, 100 ppm–70 mM), citral (TRPA1, 250 ppm) or citral-isopulegol (TRPA1-TRPM8, 250 ppm–200 ppm). Fluid thickening improved the safety of swallow by 80% (*p* < 0.0001) by delaying bolus velocity by 20.7 ± 7.0% and time to laryngeal vestibule closure (LVC) by 23.1 ± 3.7%. Capsaicin 150μM or piperine 1 mM significantly improved safety of swallow by 50% (*p* < 0.01) and 57.1% (*p* < 0.01) by speeding time to LVC by 27.6% (*p* < 0.001) and 19.5% (*p* < 0.01) and bolus velocity by 24.8% (*p* < 0.01) and 16.9% (*p* < 0.05), respectively. Cinnamaldehyde-zinc shortened the P2 latency of pSEPs by 11.0% (*p* < 0.01) and reduced N2-P2 amplitude by 35% (*p* < 0.01). In conclusion, TRPV1 and TRPV1/A1 agonists are optimal candidates to develop new pharmacological strategies to promote the recovery of brain and swallow function in patients with chronic OD.

## 1. Introduction

The prevalence of oropharyngeal dysphagia (OD) is very high in older (23–51%) and neurologic (57–84%) patients and those with neurodegenerative diseases (40–81%) [1]. OD is a condition described by the WHO in the International Classification of Diseases (WHO ICD) under the category of Symptoms and Signs involving the Digestive System that has been recognized as a major geriatric syndrome and is increasing in developed countries where aging is a major sociodemographic issue [2]. Nevertheless, it is underdiagnosed in most patients, and there is still no specific pharmacological treatment.

OD treatment consists of compensatory strategies that include thickening products (TP), texture-modified diets and postural maneuvers that do not improve swallowing function [1]. Thickening products (TPs) have been used to increase bolus viscosity in order to reduce the risk of aspiration [3], and several studies show that they have a strong therapeutic effect in different phenotypes of patients with OD [4,5]. However, TPs also increase the prevalence of oropharyngeal residue and have poor palatability, resulting in poor treatment compliance (48–56%) [3,6,7].

The research on potential pharmacological treatments for OD started in the 1990s with dopamine agonists and amantadine, showing some positive results in the prevention of aspiration pneumonia (AP). However, they cause adverse gastrointestinal and neurological events that discourage its use in the treatment of OD [8,9]. Angiotensin-Converting Enzyme (ACE) inhibitors have also been used for the prevention of AP in patients with dysphagia by decreasing the degradation of bradykinin and tachykinins, including substance P (SP). However, randomized controlled trials show no evidence that ACE inhibitors prevent aspiration in patients with dysphagia [9,10]. In the last 10 years, our group has demonstrated that patients with chronic OD associated with aging or neurological diseases not only had impairment in the biomechanics of swallow response but also in the neurophysiology of swallowing [11,12,13,14,15]. We found OD patients have severe impairment in the biomechanics of swallowing with a high prevalence of efficacy and safety impairments and a severely delayed oropharyngeal swallow response (increased time to laryngeal vestibule closure (LVC) and to upper esophageal sphincter opening (UESO) [11,16,17,18] and slow bolus velocity associated with weak tongue thrust [11]). Through neurophysiological assessment, we have described severe impairment in both the afferent (sensory) and efferent (motor) pathways of swallowing measured by pharyngeal sensory evoked potentials (pSEP) to electrical stimulation and pharyngeal motor evoked potentials (pMEP) to transcranial magnetic stimulation, respectively [12,13,14]. We found a delayed latency and reduced amplitude of pSEPs and reduced cortical excitability of motor areas in older and post-stroke patients with OD [13]. In addition, they presented an increased pharyngeal sensory threshold that correlates with a reduction in the salivary neuropeptides substance P (SP) and calcitonin gene-related peptide (CGRP). This evidence indicates that stimulation of the sensory pathway could be an optimal therapeutic strategy to treat OD [15].

One of the best strategies to stimulate the oropharyngeal sensory pathway is through Transient Receptor Potential (TRP) channels. They are widely expressed in the sensory pathway of several human tissues, and their discovery and characterization by Dr. David Julius [19] and Dr. Ardam Patapoutiam was awarded with the 2021 Nobel Prize in Physiology or Medicine. These receptors have the capacity to perceive a wide spectrum of exogenous and endogenous sensory stimuli and initiate the transmission of the sensory input from the periphery to the central nervous system. In the human oropharynx and larynx, we found TRP receptors at differential locations and levels of expression: TRPV1 (TRP channel subfamily vaniloid member 1) receptors are found in the mucosa epithelial cells and sensory nerves located below the basal lamina or innervating the mucosa, showing a decreasing gradient expression, from the tongue to the epiglottis. It is activated by harmful stimuli-like temperatures above 43 °C, acid solution with a pH lower than 5.5 and chemical compounds [20]; TRPA1 (TRP channel subfamily ankyrin member 1) receptors are found in sensory nerves located below the basal lamina with similar levels of expression in all oropharyngeal areas and are activated by noxious cold temperatures (below 18 °C) and natural or synthetic irritant substances [20]; and TRPM8 receptors are also located in sensory nerve fibers below the basal lamina and are activated by non-harmful cold stimuli (temperatures between 15 and 30 °C) and refreshing chemical substances [21]. These receptors can be activated with several natural TRP agonists such as capsaicin, piperine, and menthol [22].

The therapeutic effect of TRP agonists on swallowing function has been recently studied; however, it is still not known which of these receptors (TRPV1, TRPA1, TRPM8) and agonists offers the best pharmacological profile to improve both the biomechanics and the neurophysiology of swallowing in patients with chronic OD.

The aim of this study was to compare the effect of active stimulation of TRPV1, TRPA1 and TRPM8 receptors with that of increasing bolus viscosity on the biomechanics and neurophysiology of the swallowing response in older, post-stroke and neurodegenerative disease patients with chronic OD. To do that, we re-analyzed the raw data of seven independent studies with the same experimental design performed by our group over the last 10 years [23,24,25,26,27,28].

## 2. Results

### 2.1. Demographics and Clinical Data

A total of 329 patients with chronic OD (50.7% men; 78.3 *±* 6.3 years) were evaluated using VFS (169 in the TP studies and 178 in the TRP stimulation studies). Etiology of dysphagia was: aging (34.0%), post-stroke (51.0%), and neurodegenerative diseases (15.0%). Table 1 specifies the characteristics of each group, showing that there were no statistical demographic differences between groups. Taken together, our study population is characterized by older patients with reduced functional capacity, several comorbidities and chronic OD at high risk of nutritional and respiratory complications associated with OD.

### 2.2. The Effect on Prevalence of VFS Signs

#### 2.2.1. VFS Signs of Impaired Safety of Swallow

##### Effect of Increasing Viscosity

**Figure 1:**
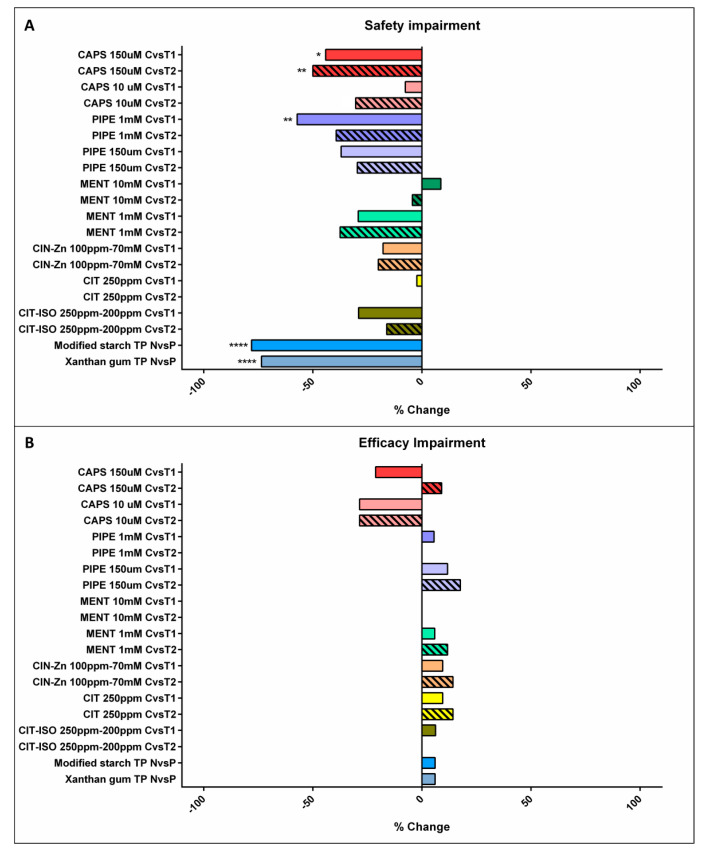
Normalized effect of compensatory and active treatments on signs of safety (**A**) and efficacy (**B**). CAPS: capsaicin; PIPE: piperine; MENT: menthol; CIN-Zn: cinnamaldehyde-zinc; CIT: citral; CIT-ISO: citral-isopulegol; C: control nectar; T1: supplemented nectar 1; T2: supplemented nectar 2; N: nectar; P: pudding; TP: thickening product; *: *p* < 0.05; **: *p* < 0.01; ****: *p* < 0.0001.

The prevalence of unsafe swallows with liquid series was 60.0%. Increasing fluid viscosity from liquid to nectar reduced the prevalence of unsafe swallows to 29.1% using XG-based TP (percentage of change from liquid of 50.3%) and to 41.4% (percentage of change from liquid of 30.2%) using MS-based TP. In addition, both types of TPs evaluated in this study caused a strong and significant reduction in the prevalence of unsafe swallows when fluid viscosity was further increased from nectar to pudding: MS TP reduced the prevalence of unsafe swallows to 9.4% (percentage of change from nectar of 84.2%), *p* < 0.0001; and XG TP to 9.7% (percentage of change from nectar of 83.4%) (*p* < 0.0001) (Figure 1a).

##### Effect of TRP Agonists

When comparing acute TRP agonist stimulation with its own non-supplemented nectar control, capsaicin significantly reduced the prevalence of safety impairment signs by 44.1% at T1 (*p =* 0.0241) and 50% (*p =* 0.0089) at T2 at a concentration of 150 µM. Piperine 1 mM also reduced the prevalence up to 57.1% at T1 (*p =* 0.0024) (Figure 1a). There were no significant differences in the therapeutic effects of TRP agonists when comparing T1 and T2. Capsaicin at a concentration of 150 µM and piperine at a concentration of 1 mM at T1 showed a stronger therapeutic effect than the other TRP agonists (Table S1).

#### 2.2.2. Efficacy Impairments

The effects of TP and TRP agonists on the efficacy of swallow are summarized in Figure 1b. Neither TRP agonists nor TPs had a significant effect on signs of impaired efficacy of swallow in this study. 

### 2.3. The Effects on the Biomechanics of the Swallow Response

#### 2.3.1. The Effect on the Timing of OSR

##### Effect of Increasing Viscosity

**Figure 2:**
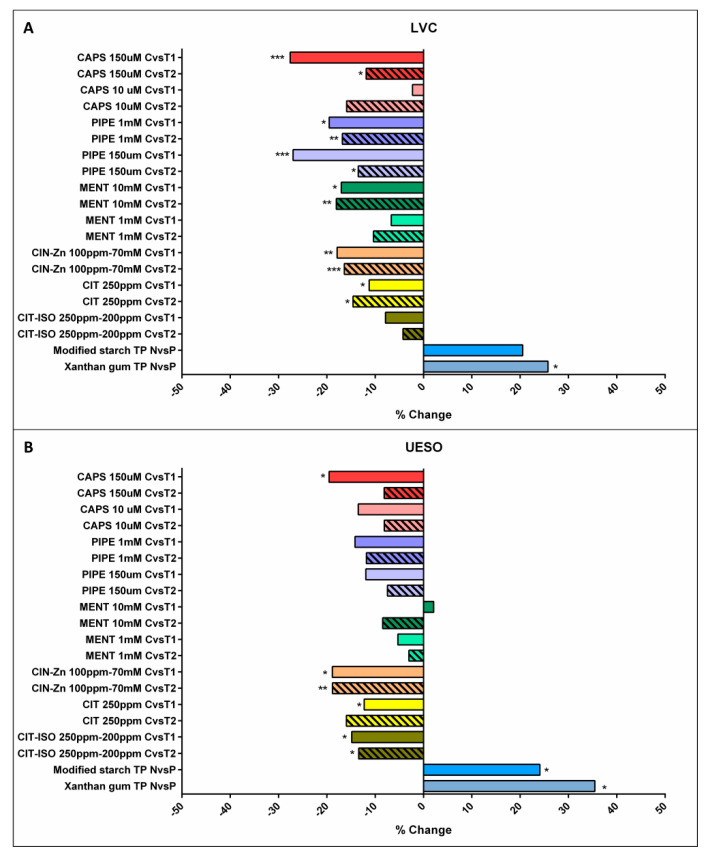
Normalized effect of compensatory and active treatments on the time to LVC (**A**) and to UESO (**B**). LVC: laryngeal vestibule closure; UESO: upper esophageal sphincter opening; CAPS: capsaicin; PIPE: piperine; MENT: menthol; CIN-Zn: cinnamaldehyde-zinc; CIT: citral; CIT-ISO: citral-isopulegol; C: control nectar; T1: supplemented nectar 1; T2: supplemented nectar 2; N: nectar; P: pudding; TP: thickening product; *: *p* < 0.05; **: *p* < 0.01; ***: *p* < 0.001.

Increasing the viscosity from nectar to pudding prolonged the timing of the OSR. MS TP significantly increased the time to UESO by 24.1% (*p =* 0.04), while XG TP increased both the time to LVC by 25.8% (*p =* 0.02) and to UESO by 35.5% (*p =* 0.03) (Figure 2a,b).

##### TRP Agonists

The three types of TRP agonists (TRPV1, TRPA1 and TRPM8 agonists) significantly reduced the time to LVC when compared with their own controls: capsaicin 150 µM (T1: 27.6%, *p =* 0.0006; T2: 11.9%, *p =* 0.0499), piperine 1 mM (T1: 19.5%, *p =* 0.0117; T2: 16.8%, *p =* 0.0061) and 150 µM (T1: 27.0%, *p =* 0.0003; T2: 13.5%, *p =* 0.0120), menthol 10 mM (T1: 17.0%, *p =* 0.0364; T2: 18.0%, *p =* 0.0102), CIN-Zn (T1: 17.9%, *p =* 0.0057; T2: 16.4%, *p =* 0.0009) and CIT (T1: 11.3%, *p =* 0.0195; T2: 14.6%, *p =* 0.0438) (Figure 2a). Capsaicin 150 µM and piperine 150 µM reduced time to LVC significantly more than menthol 1 mM, CIT and CIT-ISO (Table S2).

Regarding the time to UESO, TRPV1, TRPA1, and TRPA1-MB agonists also caused a significant reduction, the TRPV1 agonist capsaicin 150 µM reduced it by 19.5% (*p =* 0.0121) at T1, the TRPA1 agonist CIN-Zn reduced it by 18.9% (*p =* 0.0213) at T1 and 18.8% (*p =* 0.0027) at T2, the TRPA1 agonist CIT reduced it by 12.3% (*p =* 0.0309) at T1, and the TRPA1-TRPM8 agonists CIT-ISO reduced it by 14.9% (*p =* 0.0374) at T1 and 13.4% (*p =* 0.0181) at T2 (Figure 2b). When comparing between them, TRP agonists with significantly higher effect on the reduction in time to UESO were CIN-Zn, capsaicin 150 µM and CIT-ISO (Table S3).

None of the treatments showed significant differences between T1 and T2 for time to LVC or UESO.

#### 2.3.2. The Effects on Bolus Velocity

All TRP stimulants significantly increased mean bolus velocity, capsaicin 150 µM at T1 (24.8%, *p =* 0.0065), piperine 150 µM at T1 (18.3%, *p =* 0.0295), CIN-Zn at T2 (24.6%, *p =* 0.0171) and CIT-ISO at T1 (27.8%, *p =* 0.0419) and T2 (15.1%, *p =* 0.0161). In contrast, increasing bolus viscosity from nectar to pudding with modified starch and xanthan gum TP reduced bolus velocity by 25.6% and by 10.3%, respectively, but only the modified starch TP reduced it to a significant degree (*p =* 0.0319). When comparing T1 with T2, no significant differences were found.

### 2.4. The Effects on Neurophysiology

**Figure 3:**
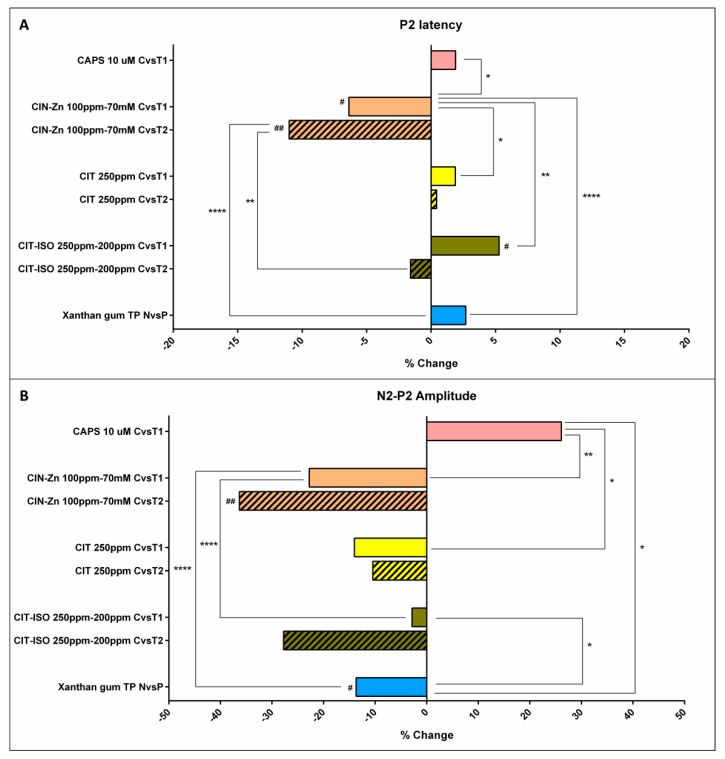
Normalized effect of compensatory and active treatments on the latency of P2 peak (**A**) and N2-P2 amplitude (**B**). CAPS: capsaicin; CIN-Zn: cinnamaldehyde-zinc; CIT: citral; CIT-ISO: citral-isopulegol; C: control nectar; T1: supplemented nectar 1; T2: supplemented nectar 2; N: nectar; P: pudding; TP: thickening product; #: *p* < 0.05; ##: *p* < 0.01 compared to its own control; *: *p* < 0.05; **: *p* < 0.01; ****: *p* < 0.0001 compared to the other treatments.

When comparing the latencies of the characteristic peaks of pSEPs before and after the acute treatments with TRP agonists, we found significant differences in P2 peak latency with CIN-Zn, which shortened it by 6.4% at T1 (*p =* 0.0454) and 11.0% at T2 (*p =* 0.0049), and CIT-ISO, which increased latency by 5.3% at T1 (*p =* 0.0188). In addition, CIN-Zn showed significant differences when compared to the other agonists and gum-based TP. In contrast, TP did not induce any significant changes in the latency of pSEPs peaks (Figure 3a).

Regarding the amplitudes, only capsaicin 10 µM showed a positive effect on N2-P2 amplitude by 26.1%, although it was not significant (*p =* 0.1563). In contrast, CIN-Zn at T2 significantly decreased it by 36.3% (*p =* 0.0049) and gum-based TP at T1 and T2 significantly decreased it by 12.5% (*p =* 0.0132) and 13.7% (*p =* 0.0407), respectively (Figure 3b).

### 2.5. Comparison on the Effectiveness of Pharmacological Treatment

#### 2.5.1. Effect on VFS Signs of Safety and Biomechanics of Swallow Response

**Figure 4:**
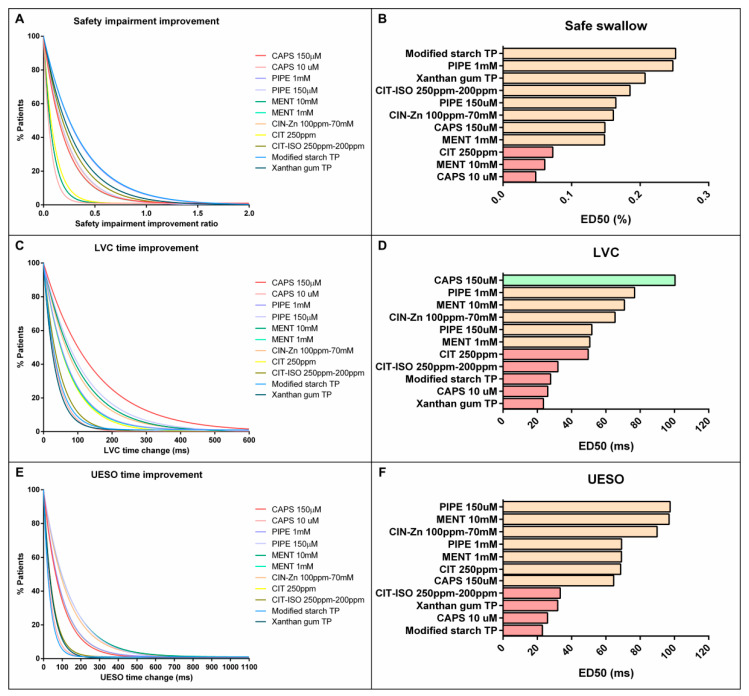
On the left, one-phase decay curves for the proportion of patients with improved signs of safety (**A**), time to LVC (**C**) and time to UESO (**E**). On the right, graphical representation of ED50 of each treatment classified as high (green bars), intermediate (orange bars) and low (red bars) therapeutic effect for signs of safety (**B**), time to LVC (**D**) and time to UESO (**F**). CAPS: capsaicin; PIPE: piperine; MENT: menthol; CIN-Zn: cinnamaldehyde-zinc; CIT: citral; CIT-ISO: citral-isopulegol; TP: thickening product; LVC: laryngeal vestibule closure; UESO: upper esophageal sphincter opening.

**Figure 5:**
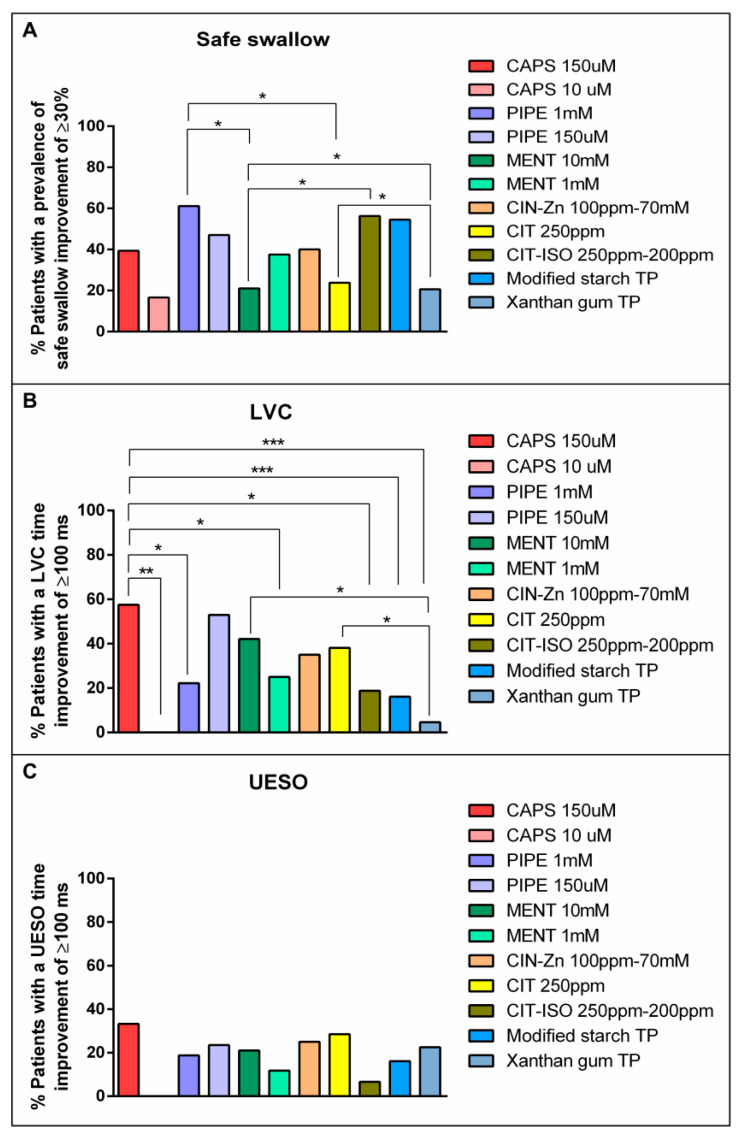
Graphical representation of percentage of patients that increased safe swallows at least 30% (**A**), reduced time to of LVC by 100 ms (**B**) and time to UESO by 100 ms (**C**). CAPS: capsaicin; PIPE: piperine; MENT: menthol; CIN-Zn: cinnamaldehyde-zinc; CIT: citral; CIT-ISO: citral-isopulegol; TP: thickening product; LVC: laryngeal vestibule closure; UESO: upper esophageal sphincter opening; *: *p* < 0.05; **: *p* < 0.01; ***: *p* < 0.001.

The one-phase decay curves showed that piperine 1 mM was the TRP agonist with higher therapeutic effect that reduced the prevalence of signs of impaired safety of swallow to a similar extent as increasing bolus viscosity with TP. However, except for capsaicin 10 µM, menthol 10 mM and CIT, which were classified in the low therapeutic effect group, most TRP agonists and TPs were classified in the intermediate therapeutic effect group (Figure 4, Table 2). In addition, piperine 1 mM was the pharmacological treatment that had the highest proportion of patients in whom the prevalence of signs of safety impairment improved by at least 30% (61.1%), which was a significant result when compared with menthol 10 mM (21.1%, *p =* 0.0201) and CIT (23.8%, *p =* 0.0253) (Figure 5).

We also observed in the time to LVC one-phase decay curve that capsaicin 150 µM had the highest therapeutic effect on airway protection mechanisms, while piperine 1 mM and 150 µM, menthol 10 mM and 1 mM and CIN-Zn had an intermediate effect and capsaicin 10 µM, CIT and CIT-ISO had the lowest one (Figure 4, Table 2). The proportion of patients treated with capsaicin 150 µM that reduced time to LVC by at least 100 ms was 57.6%. This proportion was significantly higher than patients treated with capsaicin 10 µM (0.0%, *p =* 0.0089), piperine 1 mM (22.2%, *p =* 0.0201), menthol 1 mM (25%, *p =* 0.0388), CIT-ISO (18.8%, *p =* 0.0146), starch-based TP, (16.1%, *p =* 0.0008) and gum-based TP (4.65%, *p* < 0.0001) (Figure 5). With regard to the effect on time to UESO, capsaicin 150 µM, piperine 1 mM and 150 µM, menthol 10 mM and 1 mM, CIN-Zn and CIT had an intermediate therapeutic effect, while capsaicin 10 µM, CIT-ISO and both TP types had low therapeutic effect (Figure 4, Table 2). However, no significant differences were found in the proportion of patients whose time to UESO was reduced by at least 100 ms (Figure 5).

#### 2.5.2. Effect on Neurophysiology

**Figure 6:**
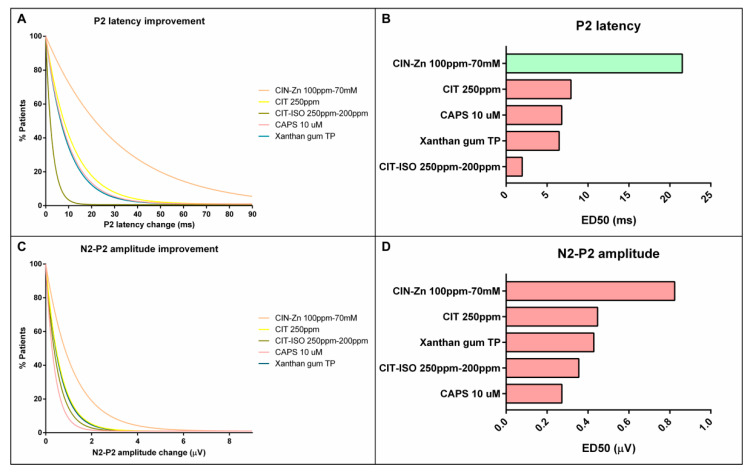
On the left, one-phase decay curves for the proportion of patients with improved P2 peak latency (**A**) and N2-P2 amplitude (**C**). In the right, graphical representation of ED50 for each treatment classified as high (green bars) and low (red bars) therapeutic effects for P2 peak latency (**B**) and N2-P2 amplitude (**D**). CAPS: capsaicin; CIN-Zn: cinnamaldehyde-zinc; CIT: citral; CIT-ISO: citral-isopulegol; TP: thickening product.

**Figure 7:**
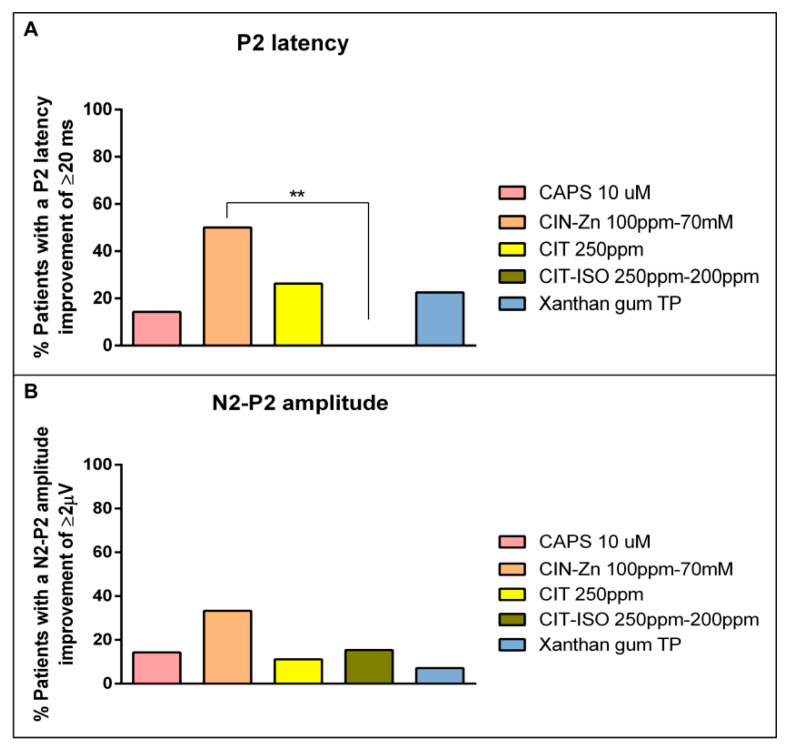
Graphical representation of percentage of patients that improved P2 peak latency (**A**) by at least 20 ms and N2-P2 amplitude (**B**) by 2µV. CAPS: capsaicin; CIN-Zn: cinnamaldehyde-zinc; CIT: citral; CIT-ISO: citral-isopulegol; TP: thickening product. **: *p* < 0.01.

Regarding the latency of P2 peak, only CIN-Zn had high therapeutic effect, while capsaicin 10 µM, CIT, XG-based TP and CIT-ISO were the agents with the lowest effect (Figure 6a,b; Table 3). The proportion of patients that improved the latency of the P2 peak after the treatment with CIN-Zn by at least 20 ms was 50.0% and only showed significant differences when compared to CIT-ISO (0.0%, *p =* 0.0052). Finally, the one-phase decay curve of N2-P2 amplitude showed that all treatments had a low pharmacological effect (Figure 6c,d; Table 3). No significant differences were found when the proportion of patients that improved by at least 2 µV was compared (Figure 7).

## 3. Discussion

This study compares the therapeutic effect of increasing fluid viscosity with two types of TP (MS, XG) versus that of six compounds of three families of TRP agonists (TRPV1, TRPA1 and TRPM8) in order to identify the optimal components to develop an active pharmacological treatment for patients with OD. The main results showed that both fluid thickening with TP and TRP agonists strongly improved the safety of swallowing through very different and even opposite mechanisms of action. While increasing shear viscosity with TP to achieve high viscosity levels (1840–3682 mPas·s, pudding) strongly protects the patients with OD from penetrations and aspirations by delaying the swallow response, reducing the mean bolus velocity in the pharynx and delaying time to LVC and UESO, TRP agonists also strongly improved the safety of swallow by speeding the swallow response, increasing bolus velocity and reducing time to LVC and UESO at much lower viscosity levels. Our study also shows TRPV1 and TRPV1/A1 agonists have the greatest therapeutic efficacy and potential to develop an active pharmacologic treatment to improve the swallowing function and restore the neurophysiology of swallowing in patients with chronic OD. An optimal strategy might be to combine these active pharmacological TRP stimulants with the compensatory effects of fluid thickening at 250 mPa·s with XG TP.

There is evidence for increasing viscosity to reduce the risk of airway invasion, and it is a valid management strategy for OD [3]. The prevalence of impaired safety of swallow (aspirations or penetrations) with thin liquid in several phenotypes of patients with OD is very high (from 50.0% in Parkinson’s disease patients to 80.6% in patients with head and neck cancer) [4,5], and the use of TPs to avoid penetrations and aspirations into the airways has become a widely used therapy for these patients with chronic OD. Many more studies than those included in this manuscript indicated that TPs had a shear viscosity-dependent effect on the safety of swallow. More recent studies established the therapeutic ranges for viscosity between 250 and 800 mPa·s, meaning that the effect on safety of swallow below 250 mPa·s is weak and that as viscosity is increased, the safety of swallow improves until a maximal therapeutic effect at 800–1000 mPa·s where further viscosity increase does not add therapeutic effect [3,4,5]. However, higher viscosities also increase the presence of oral or pharyngeal residue, especially with starch-based thickeners [3,29]. Another key element to their effectiveness is shear thinning and salivary alpha-amylase resistance, as both phenomena can cause viscosity to be significantly reduced in the oropharynx, increasing the risk of aspiration [30]. In this study, we have described the strong therapeutic effect of increasing fluid viscosity with TPs and the differences in their mechanism of action when compared with TRP agonists. TPs do not improve biomechanics nor neurophysiological parameters such as time to LVC or UESO or latency and amplitude of pSEPs. Thus, they are merely compensatory agents that do not improve swallowing physiology, forcing the patient to use these products permanently with their associated low adherence due to the low palatability, particularly for MS, especially in those patients with severe OD who require higher viscosity levels [31]. Moreover, a previous study showed that higher viscosities could even increase the time to LVC and UESO and decrease bolus velocity [28], as we also observed in the present comparative study. Some studies that have tested new generation TPs composed mainly by gums have found that the therapeutic range between the minimal and maximal protection is between 250 and 1000 mPa·s [4,5], which is a range that differs significantly from those previously used by starch-based products in which the highest viscosity was >3500 mPa·s [28]. In addition, XG TP was not significantly affected by amylase in the oral phase in contrast to 97–98% reduction in viscosity for MS TP, providing an important therapeutic advantage for these products [30].

In contrast, our study also showed how the supplementation of a nectar viscosity bolus with the TRPA1/V1 agonist piperine in a concentration of 1 mM had a similar therapeutic effect on the safety of swallow as increasing fluid thickening TP to high viscosity levels. We have found that in 50% of patients treated with piperine 1 mM, the prevalence of safety impairments was reduced by at least 25%, whereas increasing bolus viscosity with TP similarly reduced it by 20–27%. However, the effect of TRP stimulants was coupled with a significant improvement in the timing of the OSR, meaning this is an active therapy that improves swallowing physiology. As shown in our previous studies, the acute and sub-acute stimulation with TRP agonists did not only improve the PAS score but also reduced the time to UESO and to LVC [23,24,25,26,27], this last being the main mechanism that protects the respiratory airway when swallowing; LVC greater than 340 ms predicts unsafe swallow in post-stroke, Parkinson disease and dementia patients [16,17,18]. Our comparative study showed that all patients with chronic OD had a basal time to LVC greater than 340 ms and that stimulation with capsaicin (TRPV1) and piperine (TRPV1/A1), both at 150 µM, produced the greatest reduction in the time to LVC, below 340 ms cutoff, and to UESO. However, capsaicin 10 µM, menthol 1 mM and CIT-ISO were the TRP agonists with no acute effect on these parameters. As we stated in our previous studies, a lower concentration of capsaicin (10 µM) and menthol (1 mM) was not able to induce acute changes in the biomechanics or the neurophysiology of swallowing. Regarding capsaicin, we found in other studies that when treatment was maintained for 10 days, it significantly reduced the time to LVC and the PAS score [26], and it also increased the concentration of the neuropeptide substance P in saliva [32]. When comparing treatment effectiveness, capsaicin 150 µM was the TRP agonist that showed the strongest therapeutic effect on the biomechanics of the swallow response, as 50% of patients reduced the time to LVC by at least 100 ms, which is a huge biomechanical improvement on swallow physiology after TRPV1 stimulation. The mean effect of all these TRP agonists on airway protection mechanisms is: (a) a reduction of 14.4% in time to LVC, that improves safety of swallow, and (b) a similar reduction of 11.3% on time to UESO, that can improve efficiency of swallow. This might be related to the similar expression of TRPV1 and TRPA1 in the regions innervated by SLN or cranial nerve IX [20]. In addition, a study performed with an animal model has demonstrated that the application of TRPV1 and TRPM8 agonists to SLN induced a significant increase in spontaneous swallowing, and that this effect was reverted by specific antagonists, further suggesting that targeting the TRP channels could be a potential therapeutic strategy for the management of dysphagia [33]. It is also important to note that although a few patients reported some pungency during the stimulation with this high concentration of capsaicinoids, no patient dropped out of the study. This result helps us hypothesize that treatment compliance would be higher than that of the thickeners, as palatability and compliance at high viscosity levels is poor.

In a previous in vitro study using capsaicin (10^−6^ M) and piperine (10^−3^ M) in a bioassay using human PC-3 cells, we observed that repetitive stimulation with these TRP stimulants caused a reduction in the response, suggesting receptor desensitization [34]. However, in the present in vivo study, we did not observe any acute desensitization process when comparing responses to TRP agonists at T1 and T2, as the improvements in the swallowing response were maintained during the second rounds of stimulation. It has been also described that combining a TRPA1 and TRPM8 agonist can diminish the therapeutic effect they would have when applied separately because the simultaneous stimulation of these two receptors causes a cross-inhibition process [27,35], which would explain the low therapeutic effect of combined agonist CIT-ISO in our study.

Finally, the effect on the neurophysiological response showed that only CIN-Zn (TRPA1 agonist) significantly reduced the latency of P2 peak, indicating that this could improve the integration of the sensory inputs in the brain and could be associated with the improvements observed in the biomechanical response with the same agonist. This association between the improvements of both responses was observed in a previous study by our group, where we described a positive correlation between the reduction in the latency of N1 peak and the reduction in time to LVC after stimulation with capsaicin 10 µM for 10 days, suggesting that TRP stimulation induced neuroplasticity processes which were translated into biomechanical improvements [26]. In addition, acute stimulation with capsaicin also improved cortical excitability assessed with pharyngeal motor evoked potentials in post-stroke patients with OD [36]. However, all TRP agonists seemed to have a positive effect on the neurophysiological response, although they were not significant. When analyzing the effectiveness of treatments through pharmacodynamics, only CIN-Zn showed a high therapeutic effect, as in 50% of patients, the latency of P2 peak was shortened by 21 ms. In contrast, when performing the one-phase decay curve taking into account the changes induced in the N2-P2 amplitude, only capsaicin 10 µM increased it, meaning that it was improved by 2 µV in 50% of patients treated with this agonist in a low concentration.

We recognize our study has several limitations. The first one is the small sample size of some of the TRP agonists studies at the concentration tested. In addition, the only data on the effect on the neurophysiology are for capsaicin 10 µM, CIN-Zn, CIT and CIT-ISO; the effect of the other TRP agonists on the neurophysiological response was not measured. Moreover, although all studies have the same experimental design facilitating the interpretation of our results, the TPs and the viscosity used in each study were different and only data from acute studies were included, since evidence of the sub-acute effect is not yet known for all agonists. Further clinical studies are needed in order to know exactly which TRP agonist has the best mid and long-term therapeutic effect on improving the biomechanics and neurophysiology of swallowing without inducing desensitization. Finally, some basic studies are also needed to evaluate the mechanism of action of each TRP agonist.

## 4. Materials and Methods

The seven studies included in this comparison were performed in the Gastrointestinal Physiology Laboratory of the Hospital de Mataró, Catalonia, Spain. Patients treated in each study had clinical signs of chronic OD associated with aging, stroke or neurodegenerative disease. All the studies were approved by the ethics committee of the Hospital de Mataró and conducted according to the Declarations of Helsinki. Clinical trials.Gov Codes are described in Table 4.

### 4.1. Study Design

**Figure 8:**
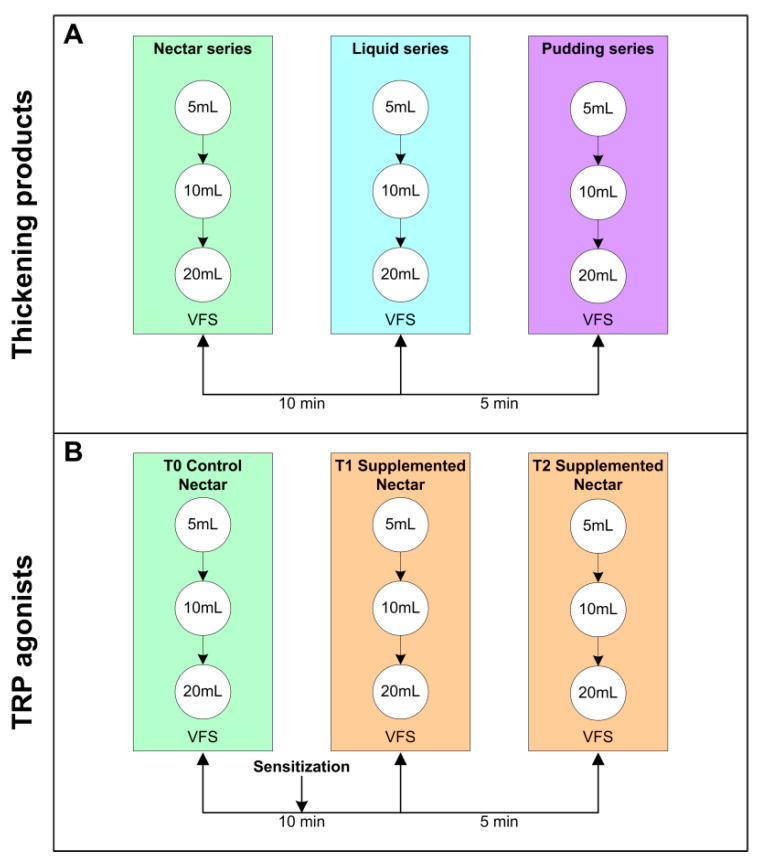
Videofluoroscopy (VFS) algorithm for each type of treatment: (**A**) thickening products and (**B**) TRP agonists.

**Figure 9:**
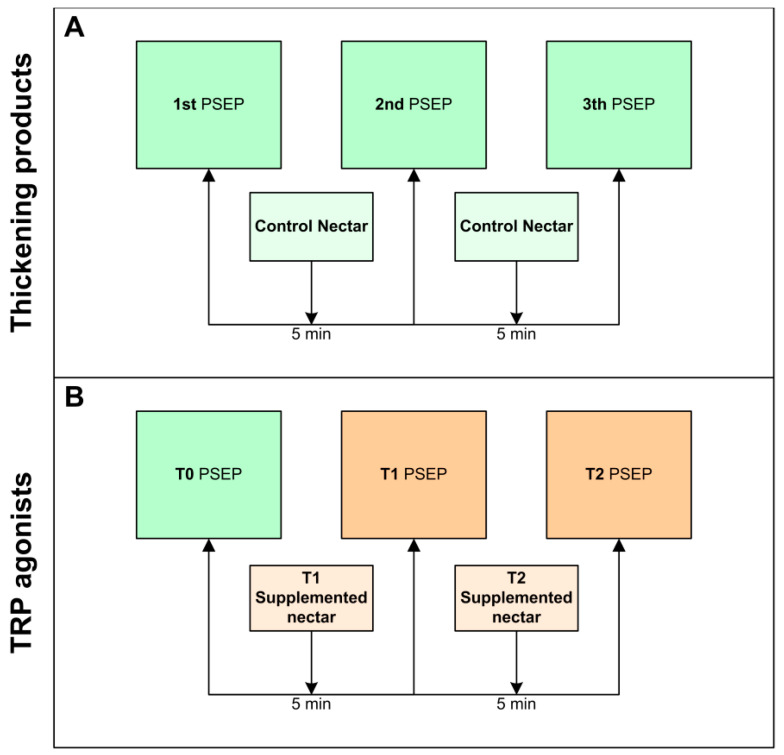
Pharyngeal sensory evoked potential (pSEP) algorithm for each type of treatment: (**A**) thickening products and (**B**) TRP agonists.

We designed an observational retrospective study to evaluate and compare the biomechanical and neurophysiological effects of TRP agonists vs. TPs. The raw data collected from all the studies included: clinical and sociodemographic data of all patients, including the cause of chronic OD and the functional (Barthel Index) and nutritional status (Mini nutritional assessment short form -MNA-sf-). The effect of TRP agonists and that of TP was assessed on the prevalence of visuoperceptual signs of impaired safety and efficacy of swallow [37] and the physiological data on biomechanical evaluation of swallow response during VFS as previously described [11,16,17]. Finally, we gathered neurophysiological information from studies that included the assessment of PSEPs to describe the effect of the treatments on swallowing neurophysiology [12,13,26,27] (Figure 8 and Figure 9, Table 4).

### 4.2. Biomechanical Assessment

#### 4.2.1. VFS Procedure

The evaluation of OD signs and swallowing biomechanics was assessed with VFS as previously described [11,16,17]. In summary, all patients were recorded in lateral projection (including the oral cavity, the pharynx, the larynx and the cervical esophagus). VFS images were obtained through a Super XT-20 Toshiba Intensifier (Toshiba Medical Systems Europe, Zoetermeer, Holland) and recorded at 25 frames/second (Ref). VFS images were recorded, digitalized and analyzed using the Swallowing Observer software (Image and Physiology SL, Barcelona, Spain). The prevalence of efficacy impairments was obtained—oral and pharyngeal residue—as well as the prevalence of safety impairments—penetrations and aspirations. In addition, several biomechanical parameters were measured while swallowing 5 mL nectar boluses: the time to laryngeal vestibule closure (LVC) (measured as the time from the opening of the glossopalatal junction (GPJO) to the LVC)), the time to upper esophageal vestibule opening (UESO) (measured as the time from the GPJO to the UESO) and mean bolus velocity in the pharynx.

#### 4.2.2. Experimental Design for VFS Studies

In independent experiments we assessed:

(a) Effect of increasing shear viscosity while swallowing 5, 10 and 20 mL of liquid, nectar (238–295 mPa·s) or pudding (1840–3682 mPa·s) viscosity series. Nectar viscosities were obtained by adding 4.5 g (295 mPa·s) of Resource ThickenUp (TU) or 1.2 g (238 mPa·s) of Resource ThickenUp Clear (TUC) to 100 mL of water. Pudding viscosities were obtained by adding 9 g (3682 mPa·s) of TU or 6 g (1840 mPa·s) of TUC to 100 mL of liquid. The liquid used was a 1:1 mixture of water and hydrosoluble radiopaque contrast (Gastrografin—Bayer Hispania SL, Barcelona, Spain for the MS thickener—or Omnipaque—GE Healthcare, Chicago, Illinois, USA- for XG). The protocol has been previously described [23,28] (Figure 8a).
(b) Effect of TRP agonists. In the acute stimulation with TRP agonists protocol, VFS consisted of a first control series (T0) of 5, 10 and 20 mL of “nectar” (238 mPas·s) boluses without supplementation followed by two 5, 10 and 20 mL nectar series (T1 and T2) supplemented with a TRP agonist. Between T0 and T1, there was a sensitization period that consisted of two 5 mL nectar boluses containing the same agonist (Figure 8b). The TRP agonists studied were: capsaicinoids (TRPV1 agonist) at 150 µM [23] and 10 µM [26], piperine (TRPV1/A1 agonist) at 1 mM and 150 µM [24], menthol (TRPM8 agonist) at 10 mM and 1 mM [25], cinnamaldehyde-zinc (CIN-Zn) at 100 ppm–70 mM (TRPA1 agonists), citral (CIT) at 250 ppm (TRPA1 agonist), and citral-isopulegol (CIT-ISO) at 250 ppm–200 ppm (TRPA1-TRPM8 agonists) [27] (Table 4).

### 4.3. Neurophysiological Assessment

#### 4.3.1. pSEPs Procedure

The pharyngeal electrical stimuli to elicit pSEPs were applied through a nasopharyngeal probe (Gaeltec Ltd., Dunvegan, Scotland) with 2 bipolar electrodes that were positioned 14–15 cm from the nostril. The probe was connected to a Digitimer DS7A current stimulator and DG2A train/delay generator (Digitimer Ltd., Welwyn Garden City, UK). Sensory (lowest intensity perceived) and tolerance (highest intensity tolerated by the patient) thresholds were determined in each patient prior to stimulation. The stimulus intensity applied to assess the pharyngeal sensory evoked potential (pSEP) was 75% of the tolerance threshold [12,13]. The cortical response to pharyngeal stimulation was recorded through a cap with 32 scalp electrodes (Electro-Cap International Inc, Eaton, OH, USA), an amplification of the 10–20 system, referenced to the left ear lobe and connected to a BrainAmp amplifier (Brain Products GmbH, Gilching, Germany). EEG was analyzed offline with BrainVision Analyzer Software 2.0 (Brain Products GmbH, Gilching, Germany) as described in our previous articles (refs). The PSEPs of each participant were recorded and averaged. The variables obtained were the latency of the peaks P1 and N1 (afferent conduction) and P2 and N2 (cortical integration) and the amplitudes P1-N1, N1-P2 and N2-P2. In this study, only the results obtained from the comparison of the latency of P2 peak and the N2-P2 amplitude before and after TRP agonist administration are shown.

#### 4.3.2. Experimental Design for pSEPs

In independent experiments, we assessed:

(a) Effect of thickeners on the neurophysiology of swallowing. A total of 3 sets of stimuli were recorded: T0 (control), T1 (first time point post-stimulation) and T2 (second time point post-stimulation). Each set was characterized by having a duration of 4:15 min in which the patient received an electrical stimulus every 5 s. There was a rest interval of 1 min between the stimulation sets, during which a single bolus of 35 mL of nectar (238 mPa·s) was taken (Figure 9a). Only the effect of XG-TP was studied as a control [27].
(b) Effect of acute stimulation with TRP agonists on the neurophysiology of swallow. The same protocol as described above was followed at T0 but with the difference that the nectar bolus taken between stimulation sets was supplemented with TRP agonists at T1 and T2. In this case, T0 was the basal situation, and T1 and T2 were used to determine the effect of TRP stimulation (Figure 9b). To avoid the effect of multiple administrations, a period of 5 days was left between the VFS and the pSEPs recording. TRP agonists studied were: capsaicinoids (TRPV1 agonist) at 10 µM [26], CIN-Zn at 100 ppm–70 mM (TRPA1 agonists), CIT at 250 ppm (TRPA1 agonist), and CIT-ISO at 250 ppm–200 ppm (TRPA1-TRPM8 agonists) [27] (Table 4).

### 4.4. Data Analysis

The raw data from the VFS and neurophysiological studies were analyzed with three different strategies:

1. Intragroup differences (effect of TRP stimulants and fluid thickening, each person acting as his own control). (a) VFS signs: the differences within the same treatment group on VFS signs of safety or efficacy of swallow were analyzed by comparing the prevalence of unsafe swallows (number of unsafe swallows/total number of swallows) or efficacy impairment signs (number of ineffective swallows/total number of swallows) of nectar series (5, 10, and 20 mL) with that of pudding for the groups treated with the thickeners, and the control nectar series T0 with T1 or T2 for the groups treated with a TRP agonist; (b) Biomechanics: the effect of increasing viscosity or TRP stimulation on the biomechanics of swallow response was assessed by comparing the time to LVC (ms) and UESO (ms) while swallowing 5 mL nectar vs. 5 mL pudding (increasing viscosity effect) and while swallowing 5 mL of control nectar (T0) vs. 5 mL nectar supplemented with a TPR agonist (T1 and T2); (c) Neurophysiology: the effect of TP and TRP stimulation on the neurophysiology of swallowing was assessed by comparing the P2 peak latency and N2-P2 amplitude of pSEPs between the control recording set (T0) vs. the first (T1) or second (T2) post-stimulation sets.
2. Intergroup differences (comparison of the therapeutic effect between all treatments, including TRP stimulation vs. fluid thickening) were assessed as follows: (a) Therapeutic effect of TP: percentage change of pudding minus nectar for each VFS sign, biomechanical and neurophysiological parameter; (b) Therapeutic effect of TRP agonists between each other: percentage change for each VFS sign, or biomechanical and neurophysiological parameter between T0 and T1 or T2; (c) Therapeutic effect of TRP agonists with TP: percentage change for each VFS sign, or biomechanical and neurophysiological parameter between T0 and T1 or T2 vs. percentage of change between nectar and pudding.
3. Pharmacodynamic effect. To normalize and facilitate the comparisons on the potency of the therapeutic effect of each compound (both TP and TRP), the acute pharmacodynamic effect of the TRP agonists and thickeners on the prevalence of safety impairments and the time to LVC and to UESO were analyzed using a one-phase decay curve (ref). These curves were constructed for each agonist and thickener with the proportion of patients whose prevalence of safety impairments improved by at least 30% and time to LVC and UESO improved at least 100 ms with the model Y = [Y0-Plateau]·e^(−K·X)^+Plateau. The same curve model was obtained for P2 latency (minimum shortening of 20 ms) and N2-P2 amplitude (increased by at least 2 µV) in order to know the effect on the neurophysiological response. In addition, we compared the proportion of patients in whom these variables improved. The curves enabled the results to be differentiated into three groups according to their pharmacological effect, taking into account the ED50 value: high, intermediate and low therapeutic effect (Table 5).

### 4.5. Statistics

Continuous variables are expressed as mean ± standard deviation (SD) and compared with unpaired *t*-test (between groups) and paired *t*-test (within groups). Categorical data are expressed as relative or relative or absolute frequencies and compared with chi-squared and Fisher’s exact tests. Non-parametric tests were used when appropriate. Statistically significant differences were considered for *p*-value < 0.05. All tests were performed using GraphPad Prism 6.0 (GraphPad Software, San Diego, CA, USA).

## 5. Conclusions

In conclusion, this comparative study showed that TRP agonists, in contrast to TPs, have the potential to induce improvements in the neurophysiology, the biomechanics and the prevalence of unsafe swallows. TRPV1 and TRPV1/A1 agonists on the one hand and XG TP at specific viscosity levels on the other seem the best candidates to develop new products for dysphagia patients by combining the strong therapeutic effects of both strategies: the intrinsic improvements of the 250 mPas·s bolus on the prevalence of safe swallows and the rehabilitation of the swallowing function induced by the TRP agonists. Understanding the specific mechanism of action of these compounds will help determine the optimal concentrations of agonists and the optimal viscosity values to reach this unmet need. The development of this new generation XG-based TP plus TRPV1 or TRPV1/A1 agonist will help to move dysphagia treatment from compensation to the recovery of the swallowing function.

## Figures and Tables

**Table 1:** Demographic characteristics of study groups. Data are presented as mean ± SD unless specifically stated.

	Total	CAPS 150 µM	CAPS 10 µM	PIPE 1 MM	PIPE 150 µM	MENT 10 mM	MENT 1 mM	CIN-ZN	CIT	CIT-ISO	MS-TP	XG-TP	*p*-Value
**n**	329	33	7	20	20	20	20	21	21	16	33	118	
**Age (years)**	78.2 ± 6.5	75.9 ± 1.9	83.5 ± 6.3	75.1 ± 3.3	76.6 ± 2.4	78.2 ± 8.2	77.6 ± 8.4	81.6 ± 7.6	78.0 ± 7.5	79.8 ± 7.9	73.9 ± 2.2	74.4 ± 12.4	>0.999
**Sex (% men)**	47.8	60.6	57.1	40	45	40	55	38.1	42.9	56.4	48.5	54.2	0.708
**Barthel Index**	71.9 ± 26.4	n/a	70 ± 33.7	74.2 ± 7.9	78.0 ± 6.9	75 ± 33.9	80.3 ± 28.5	75.8 ± 33.8	58.5 ± 30.7	63.2 ± 35.6	n/a	n/a	0.999
**MNA-sf**	11.6 ± 3.9	n/a	9.5 ± 2.9	n/a	n/a	18.0 ± 7.3	9.5 ± 2.63	n/a	n/a	n/a	n/a	9.7 ± 2.8	0.823
**OD Etiology (%)**													
Aging	41.6	30.3	57.1	60	55	n/a	n/a	62	57.1	50	30.3	34.17	0.035
Stroke	42.5	45.5	28.6	40	25	n/a	n/a	19.0	23.8	25	45.5	55	0.004
ND	15.8	24.3	14.3	0	20	n/a	n/a	19.0	19.0	25	24.2	10.83	0.018

OD: oropharyngeal dysphagia; n: number; CAPS: capsaicin; PIPE: piperine; MENT: menthol; CIN-ZN: cinnamaldehyde-zinc; CIT: citral; CIT-ISO: citral-isopulegol; ND neurodegenerative; MNA-sf: mini nutritional assessment-short form; n/a: not applicable.

**Table 2:** Parameters of the one-phase decay curves for the proportion of patients with improved prevalence of safe swallow, time to laryngeal vestibule closure and time to upper esophageal sphincter opening.

		CAPS 150 µM	CAPS 10 µM	PIPE 1 mM	PIPE 150 µM	MENT 10 mM	MENT 1 mM	CIN-Zn	CIT	CIT-ISO	MS TP	XG TP
**Safe swallow**	**K**	4.73	14.75	2.80	4.21	11.53	4.74	4.34	9.68	3.74	2.75	3.34
	**Tau**	0.21	0.07	0.36	0.24	0.09	0.21	0.23	0.10	0.27	0.36	0.30
	**R^2^**	0.78	0.46	0.91	0.85	0.53	0.72	0.76	0.55	0.84	0.89	0.27
	**ED_50_**	0.15	0.05	0.25	0.16	0.06	0.15	0.16	0.07	0.19	0.25	0.21
**LVC**	**K**	0.01	0.03	0.01	0.01	0.01	0.01	0.01	0.01	0.02	0.03	0.04
	**Tau**	144.8	73.67	110.7	72.00	102.2	93.41	71.71	46.17	37.61	39.50	24.51
	**R^2^**	0.98	0.88	0.91	0.96	0.96	0.80	0.95	0.85	0.92	0.17	0.84
	**ED_50_**	100.4	26.07	76.73	51.83	70.83	50.63	65.37	49.72	32.00	27.78	23.58
**UESO**	**K**	0.01	0.03	0.01	0.01	0.01	0.01	0.01	0.01	0.02	0.03	0.08
	**Tau**	92.34	36.92	98.33	140.6	137.8	98.23	127.8	97.57	47.41	32.67	12.20
	**R^2^**	0.89	0.35	0.54	0.85	0.74	0.43	0.68	0.39	0.34	0.12	0.73
	**ED_50_**	64.58	25.96	69.15	97.48	96.89	69.09	89.89	68.61	33.35	22.98	31.88

LVC: laryngeal vestibule closure; UESO: upper esophageal sphincter opening; CAPS: capsaicin; PIPE: piperine; MENT: menthol; CIN-Zn: cinnamaldehyde-zinc; CIT: citral; CIT-ISO: citral-isopulegol; TP: thickening product; MS: modified starch; XG: xanthan gum.

**Table 3:** Parameters of the one-phase decay curves for the proportion of patients with improved P2 peak latency and N2-P2 amplitude.

		CAPS 10 µM	CIN-Zn	CIT	CIT-ISO	XG TP
**P2 latency**	**K**	0.104	0.032	0.089	0.356	0.109
	**Tau**	9.662	31.05	11.26	2.81	9.211
	**R^2^**	−0.062	0.893	0.356	0.764	0.062
	**ED_50_**	6.79	21.52	7.92	1.96	6.48
**N2-P2 amplitude**	**K**	2.582	0.8537	1.574	1.98	1.641
	**Tau**	0.387	1.171	0.635	0.505	0.609
	**R^2^**	0.467	0.710	0.679	0.801	0.856
	**ED_50_**	0.27	0.82	0.45	0.36	0.43

CAPS: capsaicin; CIN-Zn: cinnamaldehyde-zinc; CIT: citral; CIT-ISO: citral-isopulegol; XG: xanthan gum; TP: thickening product.

**Table 4:** Summary of the studies included in the comparative study.

Study	Trial Registration Code	Thickening Product	TRP Receptor	Agonist (Concentration)	VFS	pSEP
**Compensatory treatment**						
Rofes et al. 2013 [23]	ISRCTN31088564	Modified starch	n/a	n/a	+	−
Rofes et al. 2014 [28]	NCT01158313	Xanthan gum	n/a	n/a	+	−
**Active treatment**						
Rofes et al. 2013 [23]	ISRCTN31088564	Modified starch	TRPV1	Capsaicinoids (150 µM)	+	−
Rofes et al. 2014 [24]	NCT01383694	Modified starch	TRPV1/A1	Piperine (1 mM and 150 µM)	+	−
Alvarez-Berdugo et al. 2017 [25]	NCT03050957	Modified starch	TRM8	Methol (10 mM and 1 mM)	+	−
Tomsen et al. 2019 [26]	NCT01762228	Modified starch	TRPV1	Capsaicinoids (10 µM)	+	+
Tomsen et al. 2020 [27]	NCT02422576	Xanthan Gum	TRPA1	Cinnamaldehyde-Zinc (100 ppm–70 mM)	+	+
		Xanthan Gum	TRPA1	Citral (250 ppm)	+	+
		Xanthan Gum	TRPA1-TRPM8	Citral-Isopulegol (250 ppm–200 ppm)	+	+

VFS: videofluoroscopy; PSEP: pharyngeal sensory evoked potential; n/a: not applicable; +: present data; −: absent data.

**Table 5:** Criteria to determine the intensity of the therapeutic effect.

Variable	Therapeutic Effect
Signs of safety impairment	High: >30% Improvement Intermediate: 10–29% Low: <10%
Time to LVC and UESO	High: >100 ms Improvement Intermediate: 50–99 ms Low: <50 ms
P2 peak latency	High: >20 ms Improvement Intermediate: 19–10 ms Low: <10 ms
N2-P2 amplitude	High: >2 µV Improvement Intermediate: 1–2 µV Low: <1 µV

LVC: laryngeal vestibule closure; UESO: upper esophageal sphincter opening; ms: milliseconds; µV: microvolts.

## Data Availability

Not applicable.

## Supplementary Materials

The following supporting information can be downloaded at: https://www.mdpi.com/article/10.3390/ijms231810773/s1.
