# Supplementary material for: A Comparative Study on the Effect of Acute Pharyngeal Stimulation with TRP Agonists on the Biomechanics and Neurophysiology of Swallow Response in Patients with Oropharyngeal Dysphagia
Source: Int J Mol Sci. 2022 Sep 15;23(18):10773. doi: 10.3390/ijms231810773 (PMC9506471; doi:10.3390/ijms231810773)
Supplement: Supplementary file 1 [file ijms-23-10773-s001.zip › ijms-1897475-supplementary.pdf]

Table S1. Statistical results of the comparison of the therapeutic effect on videofluoroscopic signs

		CAPS 150µM		CAPS 10µM		PIPE 1mM		PIPE 150µM		MENT 10mM		MENT 1mM		CIN-Zn		CIT		CIT-ISO		MS TP	XG TP
		**T1-C**	**T2-C**	**T1-C**	**T2-C**	**T1-C**	**T2-C**	**T1-C**	**T2-C**	**T1-C**	**T2-C**	**T1-C**	**T2-C**	**T1-C**	**T2-C**	**T1-C**	**T2-C**	**T1-C**	**T2-C**	**P-N**	**P-N**
CAPS 150µM	T1-C			**		*		*		****		****		****		****		*		****	****
	T2-C				Ns		****		****		****		****		****		****		****	****	****
CAPS 10µM	T1-C					***		*		Ns		Ns		Ns		Ns		Ns		****	****
	T2-C						Ns		Ns		Ns		Ns		Ns		Ns		Ns	*	Ns
PIPE 1mM	T1-C							***		****		****		****		****		**		***	**
	T2-C								Ns		****		Ns		Ns		****		***	****	****
PIPE 150µM	T1-C									****		*		***		****		Ns		****	****
	T2-C										****		*		Ns		****		***	****	****
MENT 10mM	T1-C											****		****		****		****		****	****
	T2-C												****		**		*		Ns	****	****
MENT 1mM	T1-C													*		****		Ns		****	****
	T2-C														**		****		****	****	****
CIN-Zn	T1-C															***		Ns		****	****
	T2-C																****		Ns	****	****
CIT	T1-C																	***		****	****
	T2-C																		*	****	****
CIT-ISO	T1-C																			****	****
	T2-C																			****	****
MS TP	P-N																				Ns

CAPS: capsaicin; PIPE: piperine; MENT: menthol; CIN-Zn: cinnamaldehyde-zinc; CIT: citral; CIT-ISO: citral-isopulegol; MS TP: modified starch thickening product; XG TP: xanthan gum thickening product; C: control nectar; T1: supplemented nectar 1; T2: supplemented nectar 2; N: nectar; P: pudding; TP: thickening product; *: $p<0.05$; **: $p<0.01$; ***: $p<0.001$; ****: $p<0.0001$; Ns: not significant.

Table S2. Statistical results of the comparison of the therapeutic effect on time to laryngeal vestibule closure

		CAPS 150µM		CAPS 10µM		PIPE 1mM		PIPE 150µM		MENT 10mM		MENT 1mM		CIN-Zn		CIT		CIT-ISO		MS TP	XG TP
		T1-C	T2-C	T1-C	T2-C	T1-C	T2-C	T1-C	T2-C	T1-C	T2-C	T1-C	T2-C	T1-C	T2-C	T1-C	T2-C	T1-C	T2-C	T1-C	T2-C	P-N	P-N
CAPS	T1-C			Ns		*		Ns		Ns		**		Ns		**		****		****	**
150µM	T2-C				Ns		Ns		Ns		Ns		Ns		Ns		Ns		Ns	**	Ns
CAPS	T1-C					Ns		Ns		Ns		Ns		Ns		Ns		Ns		Ns	Ns
10µM	T2-C						Ns		Ns		Ns		Ns		Ns		Ns		Ns	*	Ns
PIPE	T1-C							*		Ns		Ns		Ns		Ns		**		****	*
1mM	T2-C								Ns		Ns		Ns		Ns		Ns		Ns	****	*
PIPE	T1-C									Ns		**		Ns		**		****		****	**
150µM	T2-C										ns		Ns		Ns		Ns		Ns	****	*
MENT	T1-C											Ns		Ns		Ns		Ns		***	*
10mM	T2-C												Ns		Ns		Ns		Ns	****	*
MENT	T1-C													Ns		Ns		Ns		***	Ns
1mM	T2-C														Ns		Ns		Ns	****	*
CIN-Zn	T1-C															Ns		Ns		****	*
	T2-C																Ns		Ns	****	*
CIT	T1-C																	Ns		****	*
	T2-C																		ns	***	*
CIT-ISO	T1-C																			****	Ns
	T2-C																			*	Ns
MS TP	P-N																				Ns

CAPS: capsaicin; PIPE: piperine; MENT: menthol; CIN-Zn: cinnamaldehyde-zinc; CIT: citral; CIT-ISO: citral-isopulegol; MS TP: modified starch thickening product; XG TP: xanthan gum thickening product; C: control nectar; T1: supplemented nectar 1; T2: supplemented nectar 2; N: nectar; P: pudding; TP: thickening product; *: $p<0.05$; **: $p<0.01$; ***: $p<0.001$; ****: $p<0.0001$; Ns: not significant.

Table S3. Statistical results of the comparison of the therapeutic effect on time to upper esophageal sphincter opening.

		CAPS 150µM		CAPS 10µM		PIPE 1mM		PIPE 150µM		MENT 10mM		MENT 1mM		CIN-Zn		CIT		CIT-ISO		MS TP	XG TP
		**T1-C**	**T2-C**	**T1-C**	**T2-C**	**T1-C**	**T2-C**	**T1-C**	**T2-C**	**T1-C**	**T2-C**	**T1-C**	**T2-C**	**T1-C**	**T2-C**	**T1-C**	**T2-C**	**T1-C**	**T2-C**	**P-N**	**P-N**
CAPS	T1-C			Ns		Ns		Ns		*		**		Ns		Ns		Ns		****	*
150µM	T2-C				Ns		Ns		Ns		Ns		Ns		*		Ns		Ns	****	*
CAPS	T1-C					Ns		Ns		Ns		Ns		Ns		Ns		Ns		*	Ns
10µM	T2-C						Ns		Ns		Ns		Ns		****		Ns		*	****	*
PIPE	T1-C							Ns		Ns		Ns		Ns		Ns		Ns		****	*
1mM	T2-C								ns		Ns		Ns		Ns		Ns		Ns	***	*
PIPE	T1-C									Ns		Ns		Ns		Ns		Ns		****	*
150µM	T2-C										Ns		Ns		****		Ns		**	****	*
MENT	T1-C											Ns		Ns		Ns		Ns		*	ns
10mM	T2-C												Ns		****		Ns		*	****	*
MENT	T1-C													Ns		Ns		*		****	Ns
1mM	T2-C														Ns		Ns		Ns	Ns	Ns
CIN-Zn	T1-C															Ns		Ns		****	*
	T2-C																Ns		*	****	*
CIT	T1-C																	Ns		****	*
	T2-C																		Ns	****	*
CIT-ISO	T1-C																			****	*
	T2-C																			****	*
MS TP	P-N																				Ns

CAPS: capsaicin; PIPE: piperine; MENT: menthol; CIN-Zn: cinnamaldehyde-zinc; CIT: citral; CIT-ISO: citral-isopulegol; MS TP: modified starch thickening product; XG TP: xanthan gum thickening product; C: control nectar; T1: supplemented nectar 1; T2: supplemented nectar 2; N: nectar; P: pudding; TP: thickening product; *: $p<0.05$; **: $p<0.01$; ***: $p<0.001$; ****: $p<0.0001$; Ns: not significant.
